# EMT in immuno-resistance

**DOI:** 10.18632/oncoscience.226

**Published:** 2015-08-31

**Authors:** Stéphane Terry, Salem Chouaib

**Affiliations:** Inserm U1186, Gustave Roussy Cancer Campus, Villejuif, France

**Keywords:** EMT, immune resistance, cytotoxic T lymphocytes, tumor plasticity, CSC

**Figure 1:**
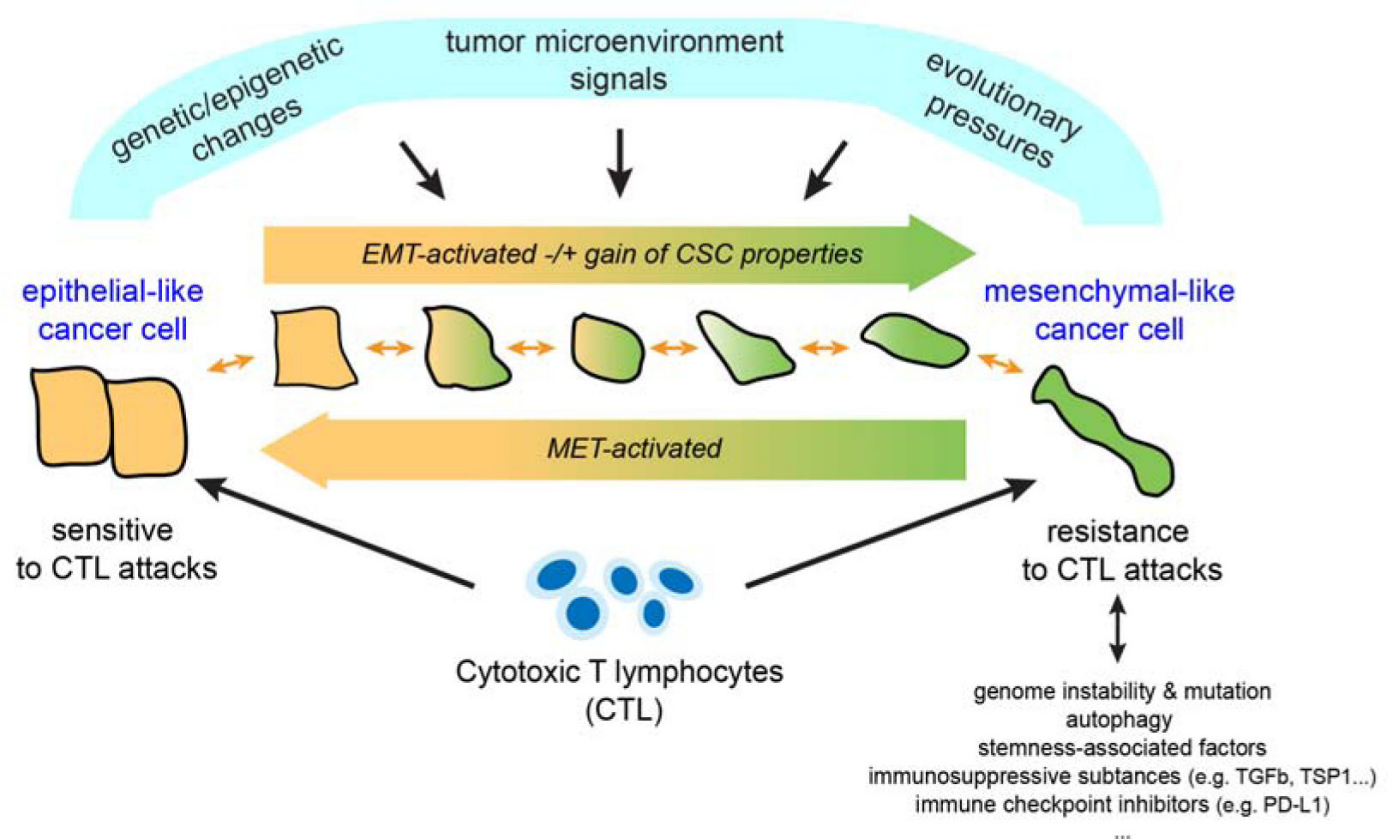
Schematic model for the emergence of EMTed tumor variants resistant to cytotoxic T lymphocytes EMT may be driven by a conjunction of environmental changes, combined with evolutionary pressures and oncogenic events emerging during tumor development. EMT may be partial or reversible with cells transitioning from an epithelial to mesenchymal state, either partially or fully, and then reverting back to a more epithelial state (MET). This reflects the plasticity of cells undergoing EMT or MET. As we now understand it, cancer cells that have acquired a more mesenchymal phenotype also show reduced susceptibility to CTL-mediated killing as well as a gain of stem-like properties. Whether these resistant variants may cooperate to escape immunosurveillance is currently unknown. Likewise, it remains unclear to what extent distinct or variable mesenchymal-like states may impact differently on susceptibility to CTL. Of note, there may be multiple rounds of EMT and MET in the life of a cancer cell (primary versus metastatic sites) potentially controlled by signals produced from different microenvironments.

Although the advent of new immunotherapy approaches has improved survival for many patients with advanced malignancies, the high degree of non-responders, especially in highly prevalent malignancies including breast, colon and prostate cancers was also a strong reminder that we possess only partial understanding of the events underlying the immune resistance of tumors. Considerable evidence indicates that the innate and adaptive immune systems participate in the recognition and destruction of cancer cells by a process known as cancer immunosurveillance. Tumor antigen-specific cytotoxic T-lymphocytes (CTL) are the major effectors in the immune response against tumor cells, and current approaches are essentially designed with the ultimate goal of inducing a strong CTL response. It is now obvious that some tumor cells can escape immunosurveillance, and accumulating evidence suggests such escape is tightly controlled by the tumor microenvironment, metabolic remodeling/hypoxia, cellular complexity and plasticity. As we develop a more complete view on the multifaceted role of tumor microenvironment in tumor development, progression, and in shaping tumor stroma, emerging observations now provide support for an essential role of tumor plasticity in resistance to CTL attacks. Along this line, epithelial mesenchymal transition (EMT) is an effective strategy by which cancer cells could gain plasticity. EMT is a transdifferentiation process whereby epithelial cells lose their epithelial properties while gaining mesenchymal properties [1]. At least a fraction of cancer cells can activate this process in response to various stimuli while they may acquire in addition a drug resistant phenotype and an increased ability to invade which is a prerequisite for entry into the circulation (as Circulating Tumor Cells) and metastatic dissemination [1]. EMT can be partial or reversible (Figure 1). This implies the existence of distinct and potentially variable phenotypic states. Moreover, activation of EMT programs in carcinoma cells can provide them with stem cell properties and as well, they should be considered as a potential source of cancer stem cells (CSCs) [2]. Several E-box binding transcription factors are known to drive EMT including SNAIL1, SNAIL2, ZEB1 and ZEB2. Exploiting the human mammary carcinoma model MCF7, we provided evidence indicating that MCF7 cells that experienced EMT after stable expression of SNAIL1, or after prolonged exposure to TNF-α exhibited reduced susceptibility to CTL-mediated lysis [3]. This appears to be coordinated with the activation of an autophagic program in these cells. Subsequent experiments targeting BECLIN1, a key component of the autophagic pathway, confirmed this observation as well as showing that impairing this autophagic state can resensitize tumor cells to CTL-induced killing. Another notable aspect of our observations is that EMTed MCF7 derivatives exhibited various mesenchymal states, as assessed by EMT scoring. The TNF-derived variant (2101) displays a relatively high EMT score compared to SNAIL1-transfected cells, which suggests the former has accumulated combinatory signals leading to a more advanced mesenchymal phenotype. Interestingly, ALDH activity, known as a mark of stemness, showed escalating levels in parallel to the estimated EMT scores, and the embryonic stem cells factors OCT4, SOX2 and NANOG were all increased in the EMTed MCF7 variants. Likewise, they had increased *in vitro* clonogenic capacity and *in vivo* tumorigenicity.

In our recent analyses, we found that silencing of WNT1-inducible signaling pathway protein 2 (thereafter WISP2) in MCF7 cells, resulted in EMT, coinciding with hyperactivity of TGF-β signaling, upregulation of stem cell factor KLF4, and impairment of CTL-mediated lysis [4]. We also noticed that EMTed MCF7 cells (shWISP2 cells) can form immunological synapses with CTL but those appeared to be less active when compared to control MCF7 cells. It is also noteworthy that blocking of TGF-β signaling in these cells using the pharmacological inhibitor A83-01, and reduction of KLF4 expression using KLF4 siRNA or by introduction of its regulatory mir-7-5p, were efficient at decreasing resistance to CTLs. This survey further indicated that WISP2 silencing had repressive effects of key presentation molecules TAP1, TAP2 and HLA-A2 representing additional ways to evade immune surveillance. This work again substantiates a link between EMT, tumor plasticity, and immune resistance. It also provides evidence that deregulation of key developmental pathways in cancer cells such as TGF-β pathway can support multiple mechanisms of immune resistance to CTL. Here, it is reassuring to realize that some of these pathways may be targetable with potential benefits for more effective therapies.

Recently various investigators pointed out the role of EMT in mounting resistance to anti-tumor immunity. Transduction of Snail in B16 melanoma cells resulted in inhibition of CTL lysis activity concomitantly with inhibition of dendritic cell maturation and expansion of suppressive Treg-like CD4+ Foxp3+ cells [5]. In various cancer models, Ricciardi et al. found that enhanced EMT features after exposure to inflammatory cytokines (i.e. TGF-β, IFN-γ and TNF-α) can impact on proliferation, differentiation and apoptosis of NK, T and B cells [6]. Chen and colleagues demonstrated in lung cancer models that downregulation of miR-200s and ZEB1 overexpression not only drive EMT but also may lead to upregulation of the programmed death 1 ligand (PD-L1) in association with exhaustion of intratumoral CD8+ T lymphocytes which ultimately promoted metastasis development [7].

While our knowledge is growing on the contribution of EMT and CSCs in cancer, it becomes clear that a better understanding of the pathways governing tumor plasticity will offer new therapeutic solutions in the future to combat cancer, predict response, and boost effectiveness of treatments. We argue that targeting carcinoma cell plasticity represents a novel strategy to better control the emergence of resistant variants. In this context, the design of innovative integrative immunotherapy approaches is warranted.
